# Antibacterial Potential of Actinomycete Extracts and Characterization of β‐Lactamase‐Producing Multidrug‐Resistant Uropathogenic *Escherichia coli*

**DOI:** 10.1002/mbo3.70143

**Published:** 2025-11-10

**Authors:** Hoda Khaledi, Nour Oude Obeid

**Affiliations:** ^1^ Iranian National Institute for Oceanography and Atmospheric Science Tehran Iran; ^2^ Department of Biology, College of Convergent Sciences and Technologies Islamic Azad University, Science and Research Branch Tehran Iran

**Keywords:** actinomycetes, antibacterial agents, multidrug‐resistant *Escherichia coli*, phylogenetic analysis, uropathogenic Infections, β‐lactamase

## Abstract

The increasing prevalence of multidrug‐resistant (MDR) uropathogenic *Escherichia coli* (UPEC) strains, particularly those producing β‐lactamase enzymes, complicates urinary tract infection (UTI) treatment and poses a significant public health threat. This study investigates the antibacterial potential of actinomycete extracts against MDR UPEC isolates while characterizing their phylogenetic diversity and β‐lactamase profiles. From 300 clinical UTI samples collected in Babil Province, Iraq, 50 UPEC isolates were confirmed via standard microbiological and biochemical assays. Phylogenetic analysis, utilizing PCR‐based detection of *chuA*, *yjaA*, and *TspE4.C2* loci, revealed that most isolates belonged to the virulent B2 group. Antibiotic susceptibility testing showed 64% of isolates were MDR, with high resistance to cephalosporins and fluoroquinolones. Screening for β‐lactamase genes identified *bla*CTX‐M‐I, *bla*TEM, and *bla*OXA as prevalent resistance determinants. Actinomycete isolates from soil samples produced ethyl acetate extracts that exhibited potent antibacterial effects against MDR UPEC, with inhibition zones exceeding 15 mm. These findings highlight actinomycetes as a promising source of novel antimicrobials to combat β‐lactamase‐producing UPEC, emphasizing the need for further compound characterization.

## Graphical abstract

Antibacterial Potential of Actinomycete Extracts Against Multidrug‐Resistant *Escherichia coli* in Babil Province, Iraq.

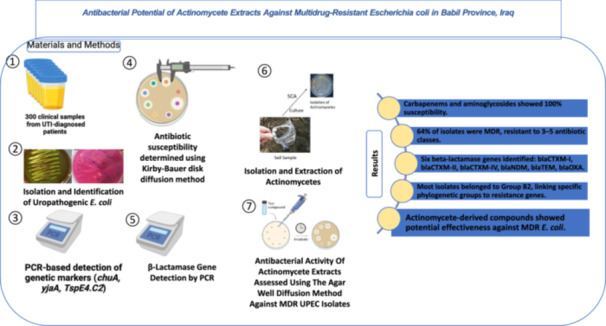

## 1 Introduction

Antimicrobial resistance (AMR) in *Escherichia coli* represents a critical global health challenge, contributing to an estimated 4.95 million deaths annually and a projected economic burden of $100 trillion by 2050 if unaddressed, according to the World Health Organization (World Health Organization 2015; O'Neill et al. 2016). The rapid dissemination of resistance genes through horizontal gene transfer, coupled with mechanisms such as extended‐spectrum β‐lactamases (ESBLs), carbapenemases, efflux pumps, and porin mutations, has rendered many conventional antibiotics ineffective (Nasrollahian et al. 2024; Pearcy et al. 2021). *E. coli*'s metabolic adaptability and transmission via the fecal‐oral route across humans, animals, and environmental reservoirs amplify its role as a major public health threat (Mączyńska et al. 2021). As a leading cause of urinary tract infections (UTIs), bloodstream infections, and other extraintestinal infections, uropathogenic *E. coli* (UPEC) employs virulence factors such as adhesins (e.g., type 1 and P fimbriae), toxins (e.g., hemolysin), and iron‐acquisition systems (e.g., siderophores), which enhance its pathogenicity and persistence in clinical settings (Čurová et al. 2020; Ou et al. 2021). The rise of multidrug‐resistant (MDR) UPEC strains, particularly those harboring *bla*CTX‐M, *bla*TEM, *bla*OXA, and *bla*NDM genes, has significantly limited therapeutic options, with resistance rates to cephalosporins and fluoroquinolones exceeding 50% in many regions, including the Middle East (Jain et al. 2020; Masoud et al. 2021; Al‐Alaq et al. 2022). High‐risk clonal lineages, such as sequence type 131 (ST131), further exacerbate resistance dissemination through mobile genetic elements like plasmids and transposons, complicating treatment strategies in both community and hospital settings (Mazumder et al. 2020).

The global AMR crisis is particularly pronounced in developing countries, where the high prevalence of MDR UPEC is driven by overuse of antibiotics, inadequate infection control, and limited surveillance systems (Cortazzo et al. 2022). In the Middle East, including Iraq, resistance to third‐generation cephalosporins and carbapenems is rising, with *bla*CTX‐M‐15 and *bla*NDM identified as dominant resistance determinants (Shrestha et al. 2023). These resistance mechanisms, often co‐occurring within single isolates, challenge the efficacy of last‐resort antibiotics like carbapenems, necessitating urgent exploration of alternative therapeutics (Zagaglia et al. 2022). The interplay of virulence and resistance in UPEC, particularly in phylogenetic group B2, underscores the need for comprehensive studies to characterize local resistance patterns and their clinical implications (Bérdy 2012).

Actinomycetes, particularly *Streptomyces* species, have been a cornerstone of antibiotic discovery since the 1940s, contributing over 60% of clinically used antibiotics, including streptomycin, tetracycline, and erythromycin (Mazumdar et al. 2023; Rozirwan et al. 2020). Sourced from diverse ecosystems such as marine sediments and terrestrial soils, actinomycetes produce a vast array of secondary metabolites with antimicrobial, anti‐biofilm, and antioxidant properties, driven by biosynthetic gene clusters like polyketide synthase (PKS) and non‐ribosomal peptide synthetase (NRPS) (El‐Sayed et al. 2022; Jagannathan et al. 2021). Recent studies have reported minimum inhibitory concentrations of actinomycete extracts ranging from 62.5 to 250 μg/mL against MDR pathogens, highlighting their potential against resistant Gram‐negative bacteria like *E. coli* (Schmiemann et al. 2010). Despite their promise, the application of actinomycete‐derived compounds against MDR UPEC remains underexplored, particularly in regions like Iraq, where environmental biodiversity may yield novel bioactive molecules (Lupindu 2017).

This study provides a novel contribution by investigating the antibacterial potential of indigenous actinomycetes from Babil Province, Iraq, against MDR UPEC, a region with limited prior research on this topic. By characterizing specific blaCTX‐M, blaTEM, blaOXA, and blaNDM resistance gene profiles and their association with phylogenetic group B2, we address a critical gap in understanding local resistance patterns. The exploration of locally sourced actinomycetes, particularly from Iraq's unique environmental niches, offers a unique perspective on combating MDR UPEC in a high‐prevalence setting, contributing to global efforts to identify alternative therapeutics.

This study aims to (i) determine the prevalence of multidrug resistance in UPEC isolates, (ii) identify β‐lactamase resistance gene profiles and phylogenetic diversity, and (iii) evaluate the bioactivity of actinomycete metabolites against MDR *E. coli* isolates to inform novel therapeutic strategies.

## 2 Materials and Methods

### 2.1 Study Design and Sample Collection

This cross‐sectional study involved 300 midstream urine samples collected between January 2022 and March 2023 from UTI patients attending three hospitals in Babil Province, Iraq: Al‐Hilla Teaching Hospital (*n* = 120), Marjan Medical City (*n* = 90), and Babylon Maternity and Pediatric Hospital (*n* = 90). These hospitals were selected because they represent the major referral centers in the province, with high UTI caseloads and established microbiology laboratories. From the 300 samples, 50 *E. coli* isolates were confirmed (20 from Al‐Hilla Teaching Hospital, 15 from Marjan Medical City, and 15 from Babylon Maternity and Pediatric Hospital) and used for subsequent analysis (Clermont et al. 2000).

### 2.2 Isolation and Identification of Uropathogenic *E. coli*

Urine samples (*n* = 300) were cultured on MacConkey and eosin methylene blue (EMB) agar (Merck, Germany) and incubated at 37°C for 24 h. From these, 50 isolates were confirmed as uropathogenic *E. coli* and were used for subsequent susceptibility testing, resistance gene detection, phylogenetic typing, and antibacterial screening. Colonies with a metallic sheen on EMB and pink, lactose‐fermenting colonies on MacConkey were selected. Confirmation of *E. coli* identity was performed using a combination of biochemical tests and molecular methods. Biochemical tests included oxidase (negative), catalase (positive), indole (positive), methyl red (positive), Voges‐Proskauer (negative), and citrate utilization (negative). Additionally, 16S rRNA sequencing was conducted on 10% of isolates (5/50) to validate identification, using universal primers 27F (5′‐AGAGTTTGATCMTGGCTCAG‐3′) and 1492R (5′‐TACGGYTACCTTGTTACGACTT‐3′). PCR amplification was performed with an initial denaturation at 95°C for 5 min, followed by 30 cycles of 95°C for 30 s, 55°C for 30 s, and 72°C for 90 s, with a final extension at 72°C for 7 min. Sequences were analyzed using BLAST against the NCBI database, confirming 99%–100% identity with *E. coli*. Isolates were stored in glycerol stocks at −20°C (CLSI 2021).

### 2.3 Phylogenetic Grouping by PCR

Genomic DNA was extracted using a commercial kit (Qiagen, Germany). Multiplex PCR targeted *chuA*, *yjaA*, and *TspE4.C2* genes to assign isolates to phylogenetic groups A, B1, B2, or D, following Clermont et al. (Magiorakos et al. 2012). PCR reactions (25 μL) contained 2.5 μL 10× PCR buffer, 200 μM dNTPs, 1.5 mM MgCl2, 1 μM primers, 1 U Taq polymerase, and 50 ng DNA. Conditions included 95°C for 5 min, 30 cycles of 94°C for 30 s, 55°C for 30 s, and 72°C for 1 min, with a final extension at 72°C for 10 min.

### 2.4 Antibiotic Susceptibility Testing

The Kirby–Bauer disk diffusion method on Mueller–Hinton agar was used, following CLSI 2021 guidelines (Mondal and Thomas 2022). Antibiotics tested included imipenem (10 μg), doripenem (10 μg), meropenem (10 μg), amikacin (30 μg), gentamicin (10 μg), nalidixic acid (30 μg), levofloxacin (5 μg), ciprofloxacin (5 μg), aztreonam (30 μg), doxycycline (30 μg), minocycline (30 μg), cefepime (30 μg), ceftazidime (30 μg), cefotaxime (30 μg), cefuroxime (30 μg), trimethoprim (5 μg), azithromycin (15 μg), and nitrofurantoin (300 μg). Testing was performed in triplicate, and results are reported as the mean percentage of susceptible, resistant, and intermediate isolates, with standard deviation calculated to assess variability. MDR was defined as resistance to three or more antibiotic classes (Kania Tri Putri et al. 2020).

### 2.5 Detection of β‐Lactamase Genes by PCR

PCR targeted *bla*CTX‐M‐I, *bla*CTX‐M‐II, *bla*CTX‐M‐IV, *bla*NDM, *bla*TEM, and *bla*OXA genes using primers listed in Table 1. Reactions (25 μL) included 2.5 μL 10× PCR buffer, 200 μM dNTPs, 1.5 mM MgCl2, 1 μM primers, 1 U Taq polymerase, and 50 ng DNA. Conditions were optimized for each gene, and products were visualized on 1.5% agarose gels with ethidium bromide.

**Table 1:** Primer sequences for detection of β‐lactamase and phylogenetic group genes in *Escherichia coli*.

Gene target	Primer sequence (5′ to 3′)	Product size	Reference
*bla*CTX‐M‐I	F: GACGATGTCACTGGCTGAGC R: AGCCGCCGACGCTAATACA	499 bp	(Aktas et al. 2012)
*bla*CTX‐M‐IV	F: GCTGGAGAAGAAAAGCAGCGGAG R: GTAAGCTGACGCAACGTCTG	474 bp	(Aktas et al. 2012)
*bla*CTX‐M‐II	F: GCGACCTGGTTAACTACAATCC R: CGGTAGTATTGCCCTTAAGCC	351 bp	(Aktas et al. 2012)
*bla*NDM	F: GGTTTGGCGATCTGGTTTTC R: CGGAATGGCTCATCACGATC	621 bp	(Tacconelli et al. 2019)
*bla*TEM	F: CGTGTCGCCTTATTCCCTT R: CAGTGCTGCAATGATACCGC	723 bp	(Naziri et al. 2020)
*bla*OXA	F: TTGGTGGCATCGATTATCGG R: GAGCACTTCTTTTGTGATGGC	473 bp	(Naziri et al. 2020)
*chuA*	F: ATGGTACCGGACGAACCAAC R: TGCCGCCAGTACCAAAGACA	288 bp	(Magiorakos et al. 2012)
*yjaA*	F: CAAACGTGAAGTGTCAGGAG R: AATGCGTTCCTCAACCTGTG	211 bp	(Magiorakos et al. 2012)
*TspE4.C2*	F: CACTATTCGTAAGGTCATCC R: AGTTTATCGCTGCGGGTCGC	152 bp	(Magiorakos et al. 2012)

### 2.6 Isolation and Extraction of Actinomycetes

Soil samples from five locations in Babil Province were collected at a depth of 5–10 cm, air‐dried, and heated at 55°C for 15 min. Diluted samples were plated on ISP‐2 agar with 50 μg/mL nystatin and incubated at 28°C for 7–10 days. Morphologically distinct colonies were subcultured and stored at 4°C. Extracts were prepared by culturing isolates in ISP‐2 broth for 10 days at 28°C with shaking, followed by ethyl acetate extraction (1:1 v/v). Extracts were concentrated using a rotary evaporator and resuspended in DMSO (Pitout et al. 2004), For antibacterial testing, the final working concentration of DMSO did not exceed 1% (v/v).

### 2.7 Antibacterial Activity Assay of Actinomycete Extracts

The agar well diffusion method was used against MDR UPEC isolates (0.5 McFarland standard). Wells (6 mm) in Mueller‐Hinton agar were filled with 50 μL of extract (10 mg/mL), with DMSO as a negative control. Plates were incubated at 37°C for 24 h, and inhibition zones ≥ 15 mm were considered significant (Kontopoulou et al. 2021). For secondary screening, a subset of 10 MDR isolates was selected from the total pool of 32, based on representation of distinct resistance phenotypes and coverage of all phylogenetic groups (B2, B1, D, and A), ensuring a clinically and genetically representative sample.

## 3 Results

### 3.1 Antimicrobial Susceptibility of *E. coli* Isolates and Selection for Actinomycete Screening

**Figure 1:**
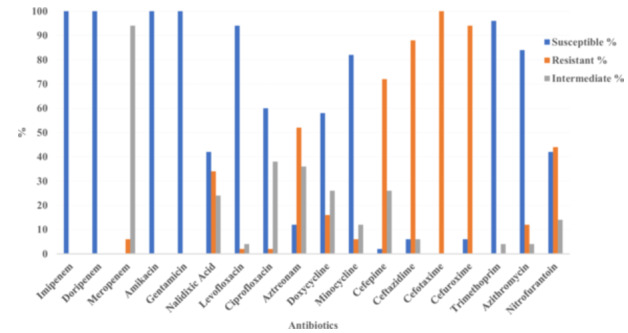
Antibiotic susceptibility, resistance, and intermediate response rates of 50 *Escherichia coli* isolates to 17 antibiotics. Data represent the mean percentage of isolates from three independent experiments, with error bars indicating standard deviation.

Of 300 urine samples, 50 *E. coli* isolates were confirmed, with 32 (64%) classified as MDR. Susceptibility testing against 18 antibiotics showed high efficacy for carbapenems (imipenem, doripenem, meropenem) and aminoglycosides (amikacin, gentamicin), with 100% susceptibility. Resistance was high for ceftazidime (88%) and cefotaxime (100%), indicating widespread cephalosporin resistance (Figure 1). In contrast, nitrofurantoin showed moderate activity, with 42% of isolates susceptible, 44% resistant, and 14% intermediate (Tables 3 and 4). These 50 isolates were subsequently subjected to primary antibacterial screening with actinomycete extracts. For secondary screening, 10 representative MDR isolates were selected from the pool of 32 MDR strains, based on their resistance profiles and phylogenetic groups, to further evaluate inhibition zone variability.

### 3.2 Multidrug Resistance (MDR) Patterns

MDR isolates exhibited resistance to three to five antibiotic classes, with monobactams, cephems, and fluoroquinolones under significant selective pressure (Table 2).

**Table 2:** MDR phenotypic resistance patterns in *Escherichia coli*.

Number of drug classes	MDR phenotype	Number of isolates	Percentage (%)
3 Drug classes	Monobactam/Cephems/Fluoroquinolone	6	40%
	Nitrofurans/Monobactam/Cephems	5	
	Monobactam/Tetracyclines/Cephems	3	
	Nitrofurans/Carbapenem/Cephems	1	
	Tetracyclines/Cephems/Nitrofurans	1	
	Nitrofurans/Fluoroquinolone/Cephems	2	
	Carbapenem/Fluoroquinolone/Cephems	1	
	Cephems/Nitrofurans/Macrolides	1	
4 Drug classes	Macrolides/Monobactam/Cephems/Fluoroquinolone	1	20%
	Monobactam/Cephems/Fluoroquinolone/Nitrofurans	1	
	Nitrofurans/Carbapenem/Cephems/Fluoroquinolone	1	
	Nitrofurans/Macrolides/Cephems/Fluoroquinolone	1	
	Monobactam/Tetracyclines/Cephems/Fluoroquinolone	1	
5 Drug classes	Nitrofurans/Monobactam/Cephems/Tetracyclines/Fluoroquinolone	2	4%

### 3.3 Beta‐Lactamase Gene Detection

**Figure 2:**
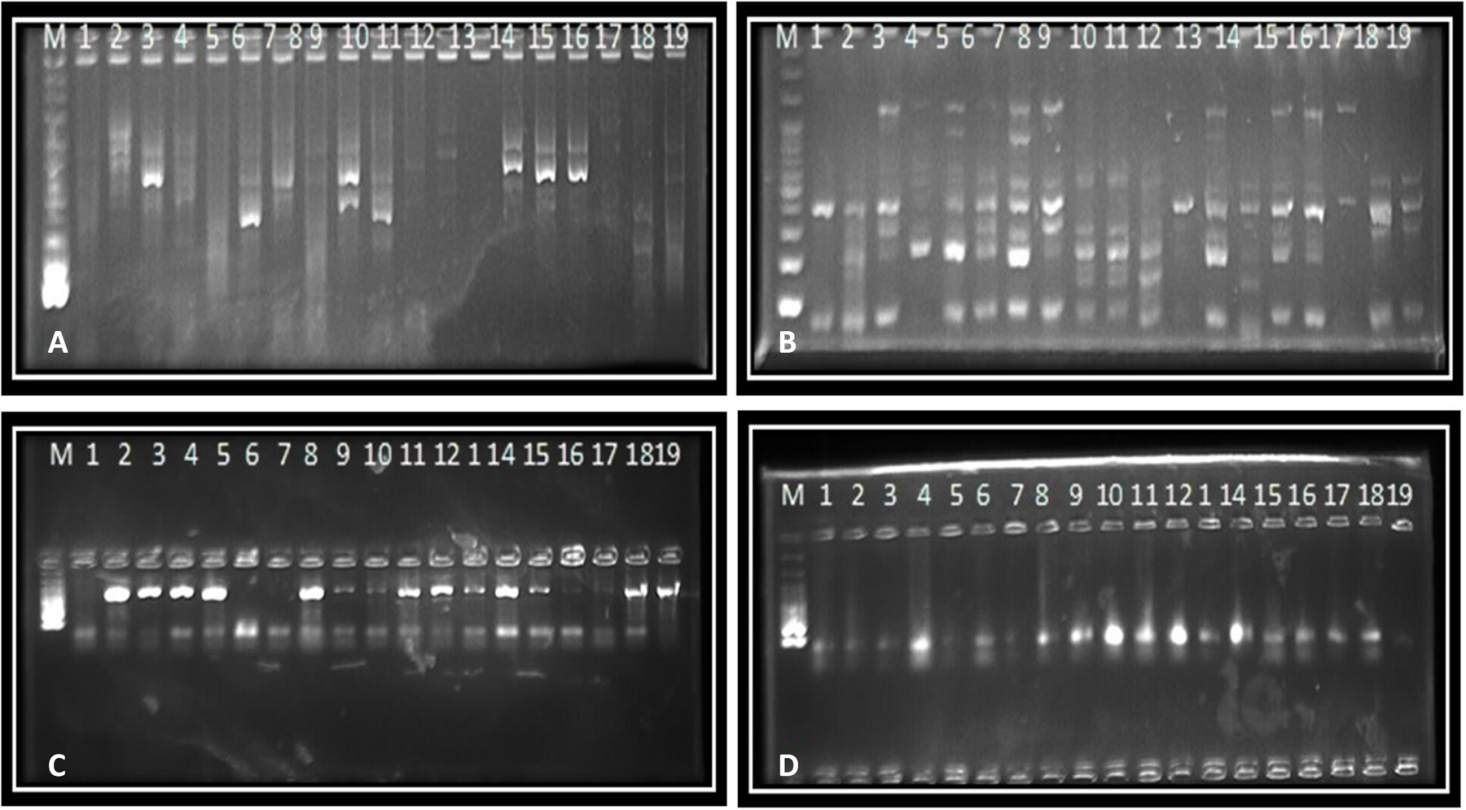
Agarose gel electrophoresis of PCR amplicons for beta‐lactamase genes in *Escherichia coli* isolates. (A) *bla*NDM (621 bp), (B) *bla*CTX‐M‐I (499 bp), (C) *bla*TEM (723 bp), (D) *bla*OXA (473 bp). Lanes: M, 100 bp DNA ladder; 1–10, representative *E. coli* isolates (E1, E2, E11, E14, E28, E29, E34, E37, E41, E50); NC, negative control (no template DNA); PC, positive control (known *E. coli* strain harboring the respective gene). Bands indicate the presence of the target gene in each isolate.

PCR detected *bla*CTX‐M‐I in 100% of isolates, *bla*CTX‐M‐II in 50%, *bla*CTX‐M‐IV in 68%, *bla*TEM in 68%, *bla*OXA in 64%, and *bla*NDM in 26%. Detailed prevalence data for *bla*CTX‐M‐II, *bla*CTX‐M‐IV, *bla*TEM, and *bla*OXA are provided in Supporting Information S1: Tables S1–S4. Agarose gel electrophoresis confirmed amplicon sizes (Figure 2).

### 3.4 Prevalence of β‐Lactamase Genes

Table 3 shows *bla*CTX‐M‐I prevalence across antibiotic susceptibility profiles, with 100% resistance to cefotaxime. Table 4 highlights *bla*NDM prevalence, with 70%–80% resistance to meropenem and nalidixic acid.

**Table 3:** Prevalence of *bla*CTX‐M‐I gene in sensitive, resistant, and intermediate *Escherichia coli* isolates.

Antibiotic	Gene status	Sensitive (S) (*N*, %)	Resistant (R) (*N*, %)	Intermediate (I) (*N*, %)
Limpenem	+	50 (100%)	0 (0%)	0 (0%)
	—	0 (0%)	0 (0%)	0 (0%)
Dorpenem	+	50 (100%)	0 (0%)	0 (0%)
	—	0 (0%)	0 (0%)	0 (0%)
Meropenem	+	0 (0%)	3 (6%)	47 (94%)
	—	0 (0%)	0 (0%)	0 (0%)
Amikacin	+	50 (100%)	0 (0%)	0 (0%)
	—	0 (0%)	0 (0%)	0 (0%)
Gentamicin	+	50 (100%)	0 (0%)	0 (0%)
	—	0 (0%)	0 (0%)	0 (0%)
Nalidixic Acid	+	21 (42%)	17 (34%)	12 (24%)
	—	0 (0%)	0 (0%)	0 (0%)
Levofloxacin	+	0 (0%)	0 (0%)	0 (0%)
	—	47 (94%)	1 (2%)	2 (4%)
Ciprofloxacin	+	30 (60%)	1 (2%)	19 (38%)
	—	0 (0%)	0 (0%)	0 (0%)
Aztreonam	+	6 (12%)	26 (52%)	18 (36%)
	—	0 (0%)	0 (0%)	0 (0%)
Doxycycline	+	29 (58%)	8 (16%)	13 (26%)
	—	0 (0%)	0 (0%)	0 (0%)
Minocycline	+	41 (82%)	3 (6%)	6 (12%)
	—	0 (0%)	0 (0%)	0 (0%)
Cefepime	+	1 (2%)	36 (72%)	13 (26%)
	—	0 (0%)	0 (0%)	0 (0%)
Ceftazidime	+	3 (6%)	44 (88%)	3 (6%)
	—	0 (0%)	0 (0%)	0 (0%)
Cefotaxime	+	0 (0%)	50 (100%)	0 (0%)
	—	0 (0%)	0 (0%)	0 (0%)
Cefuroxime	+	3 (6%)	47 (94%)	0 (0%)
	—	0 (0%)	0 (0%)	0 (0%)
Trimethoprim	+	48 (96%)	0 (0%)	2 (4%)
	—	0 (0%)	0 (0%)	0 (0%)
Azithromycin	+	42 (84%)	6 (12%)	2 (4%)
	—	0 (0%)	0 (0%)	0 (0%)
Nitrofurantoin	+	21 (42%)	22 (44%)	7 (14%)
	—	0 (0%)	0 (0%)	0 (0%)

**Table 4:** Prevalence of *bla*NDM gene in sensitive, resistant, and intermediate *Escherichia coli* Isolates.

Antibiotic	Gene Status	Sensitive (*N*, %)	Resistant (*N*, %)	Intermediate (*N*, %)
1. Imipenem	+	13 (26%)	0 (0%)	0 (0%)
	—	37 (74%)	0 (0%)	0 (0%)
2. Doripenem	+	13 (26%)	0 (0%)	0 (0%)
	—	37 (74%)	0 (0%)	0 (0%)
3. Meropenem	+	0 (0%)	2 (4%)	11 (22%)
	—	0 (0%)	1 (2%)	36 (72%)
4. Amikacin	+	13 (26%)	0 (0%)	0 (0%)
	—	37 (74%)	0 (0%)	0 (0%)
5. Gentamicin	+	13 (26%)	0 (0%)	0 (0%)
	—	37 (74%)	0 (0%)	0 (0%)
6. Nalidixic Acid	+	4 (8%)	6 (12%)	3 (6%)
	—	17 (34%)	11 (22%)	9 (18%)
7. Levofloxacin	+	11 (22%)	1 (2%)	1 (2%)
	—	36 (72%)	1 (2%)	0 (0%)
8. Ciprofloxacin	+	6 (12%)	0 (0%)	7 (14%)
	—	24 (48%)	1 (2%)	12 (24%)
9. Aztreonam	+	1 (2%)	10 (20%)	2 (4%)
	—	5 (10%)	16 (32%)	16 (32%)
10. Doxycycline	+	7 (14%)	2 (4%)	4 (8%)
	—	22 (44%)	6 (12%)	9 (18%)
11. Minocycline	+	11 (22%)	1 (2%)	1 (2%)
	—	30 (60%)	2 (4%)	5 (10%)
12. Cefepime	+	0 (0%)	12 (24%)	1 (2%)
	—	1 (2%)	24 (48%)	12 (24%)
13. Ceftazidime	+	2 (4%)	11 (22%)	0 (0%)
	—	1 (2%)	33 (66%)	3 (6%)
14. Cefotaxime	+	0 (0%)	13 (26%)	0 (0%)
	—	0 (0%)	37 (74%)	0 (0%)
15. Cefuroxime	+	0 (0%)	13 (26%)	0 (0%)
	—	3 (6%)	34 (68%)	0 (0%)
16. Trimethoprim	+	11 (22%)	0 (0%)	2 (4%)
	—	37 (74%)	0 (0%)	0 (0%)
17. Azithromycin	+	12 (24%)	1 (2%)	0 (0%)
	—	30 (60%)	5 (10%)	2 (4%)
18. Nitrofurantoin	+	3 (6%)	8 (16%)	2 (4%)
	—	18 (36%)	14 (28%)	5 (10%)

### 3.5 Phylogenetic Typing

Phylogenetic analysis classified 84% of isolates as group B2 (subgroups B22 and B23), 6% as B1, 8% as D (subgroups D1, D2), and 2% as A (Table 5).

**Table 5:** Distribution of phylogenetic groups and subgroups among *Escherichia coli* isolates.

Phylogenetic group	Subgroup	Genetic markers	No. of isolates (%)
Group A	A0	*chuA*‐/*yjaA*‐/*TspE4.C2*‐	1 (2%)
Group B1	B1	*chuA*‐/*yjaA*‐/*TspE4.C2*+	3 (6%)
Group B2	B22	*chuA*+/*yjaA*+*TspE4.C2*‐	2 (4%)
Group B2	B23	*chuA*+/*yjaA*+/*TspE4.C2*+	40 (80%)
Group D	D1	*chuA*+/*yjaA*‐/*TspE4.C2*‐	1 (2%)
Group D	D2	*chuA*+/*yjaA*‐/*TspE4.C2*+	3 (6%)

### 3.6 Antibacterial Activity of Actinomycete Extracts

Six crude actinomycete extracts were initially screened, with isolates 2–6 producing inhibition zones > 15 mm and isolate no. 3 showing the highest activity (27 mm) (Table 6). Based on our predefined criterion of ≥ 15 mm as indicative of strong activity (Section 2.7), isolate no. 3 was selected for secondary screening against 10 MDR *E. coli* isolates representing different resistance profiles and phylogenetic groups (Table 7). The use of crude extracts confirms that the antibacterial effect was mediated by secondary metabolites rather than the live actinomycete cells. In the secondary screening, inhibition zones ranged from 5 to 25 mm, with isolates E28 and E14 showing the highest susceptibility (25 mm and 22 mm, respectively). Extract from isolate no. 3 exhibited potent activity against MDR *E. coli* strains harboring blaCTX‐M‐I (100%), blaTEM (68%), and blaOXA (64%), with inhibition zones of 22–27 mm, suggesting strong efficacy against ESBL‐producing strains. Importantly, isolates E28 and E14, which co‐harbored blaCTX‐M‐I, blaTEM, and blaOXA, displayed the largest inhibition zones, indicating that the extract's activity was not compromised by these resistance mechanisms.

**Table 6:** Antibacterial activity of actinomycete isolates against *Escherichia coli*.

Actinomycete isolate no.	Inhibition zone diameter (mm)
1	5
2	15
3	27
4	18
5	14
6	17

**Table 7:** Inhibition zone diameter of actinomycete isolates against MDR *Escherichia coli*.

MDR *E. coli* isolate number	Inhibition zone diameter (mm)
E1	15
E2	17
E11	9
E14	22
E28	25
E29	15
E34	17
E37	18
E41	14
E50	5

## 4 Discussion

The 64% MDR prevalence among UPEC isolates in this study aligns with global trends, reflecting intense antibiotic selection pressure in hospital and community settings (Nordmann et al. 2011). This rate is comparable to reports from other Middle Eastern countries, such as Iran (62%) and Egypt (68%), but higher than in developed regions like Europe (30%–40%) and North America (25%–35%), where stricter antibiotic stewardship and surveillance systems are in place (Clermont et al. 2013; Farzi et al. 2021; Bush and Bradford 2020). The universal presence of *bla*CTX‐M‐I (100%) and high prevalence of *bla*TEM (68%) and *bla*OXA (64%) in our isolates mirror findings from regional studies, where *bla*CTX‐M‐15 is the dominant ESBL variant, driven by plasmid‐mediated dissemination (Muriuki et al. 2022). The detection of *bla*NDM in 26% of isolates is particularly alarming, as carbapenem resistance remains relatively low globally (5–10%) but is rising in the Middle East and South Asia due to overuse of last‐resort antibiotics and poor infection control practices (Genilloud 2017; Logan and Weinstein 2017). Our findings underscore the urgent need for stringent antibiotic stewardship programs in Iraq to mitigate the spread of carbapenemase‐producing UPEC, as well as enhanced surveillance to track emerging resistance trends (Al‐Bayati and Abed 2021).

The efficacy of actinomycete isolate no. 3 against MDR UPEC, particularly isolates harboring *bla*CTX‐M‐I, *bla*TEM, and *bla*OXA, suggests that its bioactive compounds may target mechanisms beyond β‐lactamase activity, such as cell wall disruption, efflux pump inhibition, or quorum sensing disruption, which are common in ESBL‐producing pathogens (Rybak et al. 2022). The large inhibition zones (22–27 mm) observed against isolates with complex resistance profiles, like E28 and E14, indicate that the extract's activity is not hindered by these resistance mechanisms. This broad‐spectrum activity is promising, as it suggests potential utility against a wide range of resistant pathogens, including those with multiple co‐occurring resistance genes. Notably, the extract's performance against *bla*NDM‐positive isolates (e.g., E14) is encouraging, given the limited therapeutic options for carbapenem‐resistant infections. However, further studies are needed to confirm the extract's efficacy against a larger panel of carbapenemase‐producing strains. Compared to other natural product studies, such as those reporting inhibition zones of 10–20 mm for plant‐derived antimicrobials against MDR *E. coli*, the actinomycete extract's superior activity (up to 27 mm) highlights its potential as a standout candidate for further development (Khan et al. 2009).

The phylogenetic predominance of group B2 isolates (84%) in our study aligns with global reports linking B2 to enhanced virulence and resistance in UPEC, particularly the ST131 lineage (Al‐Khafaji and Al‐Thahab 2017). The association between group B2 and *bla*CTX‐M‐I/*bla*TEM suggests that resistance plasmids are circulating within this high‐risk lineage, likely facilitated by horizontal gene transfer via conjugative plasmids or integrons (Jamali et al. 2020). This finding has significant implications for infection control, as B2 strains are more likely to cause severe, recurrent UTIs and extraintestinal infections, potentially leading to increased morbidity and healthcare costs (Akya et al. 2019). The presence of subgroups B22 and B23, associated with extraintestinal pathogenic *E. coli* (ExPEC), further underscores the pathogenic potential of these isolates and their role in community‐acquired and nosocomial infections (Barka et al. 2016). In contrast, regions like East Asia report a higher prevalence of group D (20%–30%), suggesting regional variations in UPEC population structures that warrant further comparative genomic studies (Yamaji et al. 2018).

From a public health perspective, the high MDR prevalence and *bla*NDM detection in this study signal a growing threat to UTI management in Iraq. The co‐occurrence of ESBL and carbapenemase genes in UPEC isolates could lead to treatment failures, prolonged hospital stays, and increased reliance on toxic or less effective drugs like colistin, which carries risks of nephrotoxicity and resistance development (Falagas et al. 2005). Actinomycete extracts offer a potential lifeline, as their efficacy against MDR UPEC could reduce dependence on last‐resort antibiotics and provide a sustainable alternative in resource‐limited settings. Moreover, the environmental sourcing of actinomycetes from Iraqi soils highlights the untapped potential of local biodiversity, which could be leveraged to address regional AMR challenges while contributing to global antibiotic discovery efforts (Abdelmohsen et al. 2010).

A major limitation of this study is the lack of chemical characterization of the active compounds in actinomycete extracts, particularly isolate no. 3, which showed the highest antibacterial activity. Identifying the specific bioactive metabolites (e.g., polyketides, flavonoids, or novel peptides) responsible for the observed inhibition is critical for therapeutic development and understanding their mode of action. Additionally, the study's sample size (50 isolates) may not fully capture the diversity of resistance mechanisms in Babil Province, let alone Iraq as a whole, and the focus on a single province limits generalizability to other regions with potentially different resistance profiles. The absence of in vivo testing further restricts conclusions about clinical efficacy, as factors like bioavailability, toxicity, and host immune interactions remain unassessed. Future research should employ advanced techniques such as liquid chromatography‐mass spectrometry (LC‐MS) and nuclear magnetic resonance (NMR) to elucidate the chemical composition and mechanisms of action of these extracts, alongside genomic sequencing to identify biosynthetic gene clusters responsible for their production (Challis 2008). In vivo studies in animal models of UTI are also essential to validate therapeutic potential and assess pharmacokinetics. Expanding the study to multiple regions in Iraq, and incorporating longitudinal surveillance, would provide a more comprehensive understanding of local resistance patterns and actinomycete biodiversity, strengthening the case for their development as novel therapeutics (Cantas et al. 2013). In addition, soil pH—an important factor influencing actinomycete diversity—was not measured at the time of collection. Future studies should incorporate physicochemical soil parameters to provide better context for microbial diversity and metabolite production.

The promising antibacterial activity of actinomycete extracts, particularly against MDR UPEC, underscores the potential of natural products as a source of novel antimicrobials in an era of declining antibiotic discovery (Rajivgandhi et al. 2016). Compared to synthetic antibiotics, actinomycete‐derived compounds benefit from evolutionary optimization in microbial ecosystems, potentially offering unique mechanisms of action less prone to cross‐resistance (Wright 2017). Our findings contribute to the growing body of evidence supporting the use of actinomycete‐derived compounds against resistant Gram‐negative pathogens, aligning with global initiatives like the WHO's Global Action Plan on AMR to innovate new treatment options (World Health Organization 2015). By tapping into Iraq's microbial resources, this study not only addresses a local public health crisis but also enriches the global pipeline of antibiotic candidates (Kotwani et al. 2021).

## 5 Conclusion

This study revealed high MDR prevalence (64%) among UPEC isolates, with *bla*CTX‐M‐I, *bla*TEM, and *bla*OXA as key resistance determinants. Phylogenetic group B2 dominated, suggesting a link between virulence and resistance. Actinomycete extracts, particularly isolate no. 3, showed potent activity against MDR UPEC, offering a promising alternative to conventional antibiotics. Further characterization of these compounds is warranted to develop novel therapeutics.

## Author Contributions

**Hoda Khaledi:** writing – review and editing, writing – original draft, supervision, data curation, conceptualization, resources, visualization. **Nour Oude Obeid:** methodology, software, investigation, formal analysis, validation, project administration.

## Ethics Statement

This study was approved by the Institutional Review Board of the College of Medicine, University of Babylon, Iraq (Approval No. IRB‐2021‐034). Informed consent was obtained from all participants, and the study was conducted in accordance with the Declaration of Helsinki and relevant institutional regulations.

## Conflicts of Interest

The authors declare no conflicts of interest.

## Supporting information


**Table S1:** Prevalence of blaCTXM‐I Gene in Sensitive, Resistant and Intermediate *E. coli.* **Table S2:** Prevalence of blaCTXM‐IV gene in sensitive and resistant *E. col*i isolates. **Table S3:** Prevalence of the blaTEM gene in sensitive and resistant *E. coli* isolates. **Table S4:** Prevalence of the blaOXA gene in sensitive and resistant *E. coli* isolates.

## Data Availability

All data generated or analyzed during this study are included in this published article (and its supplementary information files).
