# Supplementary material for: Antibacterial Potential of Actinomycete Extracts and Characterization of β‐Lactamase‐Producing Multidrug‐Resistant Uropathogenic Escherichia coli
Source: Microbiologyopen. 2025 Nov 10;14(6):e70143. doi: 10.1002/mbo3.70143 (PMC12602258; doi:10.1002/mbo3.70143)
Supplement: Supplementary file 1 — Table S1: Prevalence of blaCTXM‐I Gene in Sensitive, Resistant and Intermediate E. coli. Table S2: Prevalence of blaCTXM‐IV gene in sensitive and resistant E. coli isolates. Table S3: Prevalence of the blaTEM gene in sensitive and resistant E. coli isolates. Table S4: Prevalence of the blaOXA gene in sensitive and resistant E. coli isolates. [file MBO3-14-e70143-s001.docx]

**Table S1: Prevalence of blaCTXM-I Gene in Sensitive, Resistant and Intermediate *E. coli* Isolates**

| Antibiotic | Gene Status | Sensitive (S) (N, %) | Resistant (R) (N, %) | Intermediate (I) (N, %) |
| --- | --- | --- | --- | --- |
| Limpenem | + | 50 (100%) | 0 (0%) | 0 (0%) |
|  | - | 0 (0%) | 0 (0%) | 0 (0%) |
| Dorpenem | + | 50 (100%) | 0 (0%) | 0 (0%) |
|  | - | 0 (0%) | 0 (0%) | 0 (0%) |
| Meropenem | + | 0 (0%) | 3 (6%) | 47 (94%) |
|  | - | 0 (0%) | 0 (0%) | 0 (0%) |
| Amikacin | + | 50 (100%) | 0 (0%) | 0 (0%) |
|  | - | 0 (0%) | 0 (0%) | 0 (0%) |
| Gentamicin | + | 50 (100%) | 0 (0%) | 0 (0%) |
|  | - | 0 (0%) | 0 (0%) | 0 (0%) |
| Nalidixic Acid | + | 21 (42%) | 17 (34%) | 12 (24%) |
|  | - | 0 (0%) | 0 (0%) | 0 (0%) |
| Levofloxacin | + | 0 (0%) | 0 (0%) | 0 (0%) |
|  | - | 47 (94%) | 1 (2%) | 2 (4%) |
| Ciprofloxacin | + | 30 (60%) | 1 (2%) | 19 (38%) |
|  | - | 0 (0%) | 0 (0%) | 0 (0%) |
| Aztreonam | + | 6 (12%) | 26 (52%) | 18 (36%) |
|  | - | 0 (0%) | 0 (0%) | 0 (0%) |
| Doxycycline | + | 29 (58%) | 8 (16%) | 13 (26%) |
|  | - | 0 (0%) | 0 (0%) | 0 (0%) |
| Minocycline | + | 41 (82%) | 3 (6%) | 6 (12%) |
|  | - | 0 (0%) | 0 (0%) | 0 (0%) |
| Cefepime | + | 1 (2%) | 36 (72%) | 13 (26%) |
|  | - | 0 (0%) | 0 (0%) | 0 (0%) |
| Ceftazidime | + | 3 (6%) | 44 (88%) | 3 (6%) |
|  | - | 0 (0%) | 0 (0%) | 0 (0%) |
| Cefotaxime | + | 0 (0%) | 50 (100%) | 0 (0%) |
|  | - | 0 (0%) | 0 (0%) | 0 (0%) |
| Cefuroxime | + | 3 (6%) | 47 (94%) | 0 (0%) |
|  | - | 0 (0%) | 0 (0%) | 0 (0%) |
| Trimethoprim | + | 48 (96%) | 0 (0%) | 2 (4%) |
|  | - | 0 (0%) | 0 (0%) | 0 (0%) |
| Azithromycin | + | 42 (84%) | 6 (12%) | 2 (4%) |
|  | - | 0 (0%) | 0 (0%) | 0 (0%) |
| Nitrofurantoin | + | 21 (42%) | 22 (44%) | 7 (14%) |
|  | - | 0 (0%) | 0 (0%) | 0 (0%) |

**Table S2: Prevalence of blaCTXM-IV gene in sensitive and resistant *E. coli* isolates**

| Antibiotic | Gene | Sensitive | | Resistant | | Intermediate | |
| --- | --- | --- | --- | --- | --- | --- | --- |
|  |  | N | % | N | % | N | % |
| Imipenem | + | 34 | 68 | 0 | 0 | 0 | 0 |
|  | - | 16 | 32 | 0 | 0 | 0 | 0 |
| Doripenem | + | 34 | 68 | 0 | 0 | 0 | 0 |
|  | - | 16 | 32 | 0 | 0 | 0 | 0 |
| Meropenem | + | 0 | 0 | 2 | 4 | 32 | 64 |
|  | - | 0 | 0 | 1 | 2 | 15 | 30 |
| Amikacin | + | 34 | 68 | 0 | 0 | 0 | 0 |
|  | - | 16 | 32 | 0 | 0 | 0 | 0 |
| Gentamicin | + | 34 | 68 | 0 | 0 | 0 | 0 |
|  | - | 16 | 32 | 0 | 0 | 0 | 0 |
| Nalidixic acid | + | 12 | 24 | 12 | 24 | 10 | 20 |
|  | - | 9 | 18 | 5 | 10 | 2 | 4 |
| Levofloxacin | + | 31 | 62 | 1 | 2 | 2 | 4 |
|  | - | 16 | 32 | 0 | 0 | 0 | 0 |
| Ciprofloxacin | + | 20 | 40 | 1 | 2 | 13 | 26 |
|  | - | 10 | 20 | 6 | 12 | 0 | 0 |
| Aztreonam | + | 5 | 10 | 19 | 38 | 10 | 20 |
|  | - | 1 | 2 | 7 | 14 | 8 | 16 |
| Doxycycline | + | 19 | 38 | 7 | 14 | 8 | 16 |
|  | - | 10 | 20 | 1 | 2 | 5 | 10 |
| Minocycline | + | 30 | 60 | 3 | 6 | 1 | 2 |
|  | - | 11 | 22 | 0 | 0 | 5 | 10 |
| Cefepime | + | 0 | 0 | 26 | 52 | 8 | 16 |
|  | - | 1 | 2 | 10 | 20 | 5 | 10 |
| Ceftazidime | + | 3 | 6 | 31 | 62 | 0 | 0 |
|  | - | 0 | 0 | 13 | 26 | 3 | 6 |
| Cefotaxime | + | 0 | 0 | 34 | 68 | 0 | 0 |
|  | - | 0 | 0 | 16 | 32 | 0 | 0 |
| Cefuroxime | + | 1 | 2 | 33 | 66 | 0 | 0 |
|  | - | 2 | 4 | 14 | 28 | 0 | 0 |
| Trimethoprim | + | 32 | 64 | 0 | 0 | 2 | 4 |
|  | - | 16 | 32 | 0 | 0 | 0 | 0 |
| Azithromycin | + | 30 | 60 | 3 | 6 | 1 | 2 |
|  | - | 12 | 24 | 3 | 6 | 1 | 2 |
| Nitrofurantoin | + | 11 | 22 | 16 | 32 | 7 | 14 |
|  | - | 10 | 20 | 6 | 12 | 0 | 0 |

**Table S3: Prevalence of the blaTEM gene in sensitive and resistant *E. coli* isolates**

| Antibiotic | Gene | Sensitive | | Resistant | | Intermediate | |
| --- | --- | --- | --- | --- | --- | --- | --- |
|  |  | N | % | N | % | N | % |
| Imipenem | + | 34 | 68 | 0 | 0 | 0 | 0 |
|  | - | 16 | 32 | 0 | 0 | 0 | 0 |
| Doripenem | + | 34 | 68 | 0 | 0 | 0 | 0 |
|  | - | 16 | 32 | 0 | 0 | 0 | 0 |
| Meropenem | + | 0 | 0 | 3 | 6 | 31 | 62 |
|  | - | 0 | 0 | 0 | 0 | 16 | 32 |
| Amikacin | + | 34 | 68 | 0 | 0 | 0 | 0 |
|  | - | 16 | 32 | 0 | 0 | 0 | 0 |
| Gentamicin | + | 34 | 68 | 0 | 0 | 0 | 0 |
|  | - | 16 | 32 | 0 | 0 | 0 | 0 |
| Nalidixic acid | + | 16 | 32 | 11 | 22 | 7 | 14 |
|  | - | 5 | 10 | 6 | 12 | 5 | 10 |
| Levofloxacin | + | 32 | 64 | 0 | 0 | 2 | 4 |
|  | - | 15 | 30 | 1 | 2 | 0 | 0 |
| Ciprofloxacin | + | 19 | 38 | 0 | 0 | 15 | 30 |
|  | - | 11 | 22 | 1 | 2 | 4 | 8 |
| Aztreonam | + | 6 | 12 | 15 | 30 | 13 | 26 |
|  | - | 0 | 0 | 11 | 22 | 5 | 10 |
| Doxycycline | + | 21 | 42 | 5 | 10 | 8 | 16 |
|  | - | 8 | 16 | 3 | 6 | 5 | 10 |
| Minocycline | + | 28 | 56 | 3 | 6 | 3 | 6 |
|  | - | 13 | 26 | 0 | 0 | 3 | 6 |
| Cefepime | + | 1 | 2 | 24 | 48 | 9 | 18 |
|  | - | 0 | 0 | 12 | 24 | 4 | 8 |
| Ceftazidime | + | 2 | 4 | 30 | 60 | 2 | 4 |
|  | - | 1 | 2 | 14 | 28 | 1 | 2 |
| Cefotaxime | + | 0 | 0 | 34 | 68 | 0 | 0 |
|  | - | 0 | 0 | 16 | 32 | 0 | 0 |
| Cefuroxime | + | 2 | 4 | 32 | 64 | 0 | 0 |
|  | - | 1 | 2 | 15 | 30 | 0 | 0 |
| Trimethoprim | + | 33 | 66 | 0 | 0 | 1 | 2 |
|  | - | 15 | 30 | 0 | 0 | 1 | 2 |
| Azithromycin | + | 29 | 58 | 4 | 8 | 1 | 2 |
|  | - | 13 | 26 | 2 | 4 | 1 | 2 |
| Nitrofurantoin | + | 15 | 30 | 14 | 28 | 5 | 10 |
|  | - | 6 | 12 | 8 | 16 | 2 | 4 |

**Table S4: Prevalence of the blaOXA gene in sensitive and resistant *E. coli* isolates**

| Antibiotic | Gene | Sensitive | | Resistant | | Intermediate | |
| --- | --- | --- | --- | --- | --- | --- | --- |
|  |  | N | % | N | % | N | % |
| Imipenem | + | 32 | 64 | 0 | 0 | 0 | 0 |
|  | - | 18 | 36 | 0 | 0 | 0 | 0 |
| Doripenem | + | 32 | 64 | 0 | 0 | 0 | 0 |
|  | - | 18 | 36 | 0 | 0 | 0 | 0 |
| Meropenem | + | 0 | 0 | 2 | 4 | 30 | 60 |
|  | - | 0 | 0 | 1 | 2 | 17 | 34 |
| Amikacin | + | 25 | 50 | 0 | 0 | 0 | 0 |
|  | - | 25 | 50 | 0 | 0 | 0 | 0 |
| Gentamicin | + | 32 | 64 | 0 | 0 | 0 | 0 |
|  | - | 18 | 36 | 0 | 0 | 0 | 0 |
| Nalidixic acid | + | 15 | 30 | 10 | 20 | 7 | 14 |
|  | - | 6 | 12 | 7 | 14 | 5 | 10 |
| Levofloxacin | + | 31 | 62 | 0 | 0 | 1 | 2 |
|  | - | 16 | 32 | 1 | 2 | 1 | 2 |
| Ciprofloxacin | + | 17 | 34 | 1 | 2 | 14 | 28 |
|  | - | 13 | 26 | 0 | 0 | 5 | 10 |
| Aztreonam | + | 6 | 12 | 14 | 28 | 12 | 24 |
|  | - | 0 | 0 | 12 | 24 | 6 | 12 |
| Doxycycline | + | 19 | 38 | 4 | 8 | 9 | 18 |
|  | - | 10 | 20 | 4 | 8 | 4 | 8 |
| Minocycline | + | 28 | 56 | 2 | 4 | 2 | 4 |
|  | - | 13 | 26 | 1 | 2 | 4 | 8 |
| Cefepime | + | 1 | 2 | 22 | 44 | 9 | 18 |
|  | - | 0 | 0 | 14 | 28 | 4 | 8 |
| Ceftazidime | + | 2 | 4 | 27 | 54 | 3 | 6 |
|  | - | 1 | 2 | 17 | 34 | 0 | 0 |
| Cefotaxime | + | 0 | 0 | 32 | 64 | 0 | 0 |
|  | - | 0 | 0 | 18 | 36 | 0 | 0 |
| Cefuroxime | + | 3 | 6 | 29 | 58 | 0 | 0 |
|  | - | 0 | 0 | 18 | 36 | 0 | 0 |
| Trimethoprim | + | 32 | 64 | 0 | 0 | 0 | 0 |
|  | - | 16 | 32 | 0 | 0 | 2 | 4 |
| Azithromycin | + | 27 | 54 | 4 | 8 | 1 | 2 |
|  | - | 15 | 30 | 2 | 4 | 1 | 2 |
| Nitrofurantoin | + | 11 | 22 | 16 | 32 | 5 | 10 |
|  | - | 10 | 20 | 6 | 12 | 2 | 4 |
